# Increase of cyclooxygenase-2 inhibition with celecoxib combined with 5-FU enhances tumor cell apoptosis and antitumor efficacy in a subcutaneous implantation tumor model of human colon cancer

**DOI:** 10.1186/1477-7819-11-16

**Published:** 2013-01-24

**Authors:** De-Qing Zhang, Qiang Guo, Jian-Hong Zhu, Wei-Chang Chen

**Affiliations:** 1 Department of Digestion Internal Medicine, The First Affiliated Hospital of Soochow University, Su Zhou, JiangSu 215006, China; 2 Department of Emergency and Intensive Care Unit, The First Affiliated Hospital of Soochow University, Su Zhou, JiangSu 215006, China

**Keywords:** Celecoxib, 5-Fluorouracil, Colorectal cancer, Apoptosis

## Abstract

**Background:**

The purpose of this study was to investigate the anti-tumor effect and explore the mechanisms of celecoxib (a selective cyclooxygenase-2 inhibitor) combined with 5-fluorouracil (5-FU) on the treatment of human colorectal cancer in a BALB/C nude mouse subcutaneous xenograft model.

**Methods:**

Effects of celecoxib combined with 5-FU on the proliferation of xenograft carcinoma induced by HT-29 were investigated. The apoptotic cells were detected by electron microscope and TUNEL (terminal deoxynucleotidyl transferase dUTP nick end labeling) assay. Immunohistochemistry and Western blot were used to estimate the expression of cytochrome C, caspase-3 and caspase-9.

**Results:**

Compared with the control group, treatment groups showed significant inhibition of tumor growth. More apoptotic cells existed after treatment with celecoxib combined with 5-FU. Cytochrome C, caspase-3 and caspase-9 were increased in treated groups, and more obviously in the drug combination group. Cyclooxygenase-2 (COX-2) were decreased after treatment with celecoxib only or combined with 5-FU. And the combined group showed a greater decrease.

**Conclusions:**

Celecoxib combined with 5-FU could inhibit the growth of tumors *in vivo* by inducing apoptosis and activation of the cytochrome C dependency apoptosis signal pathway. A decrease of COX-2 and an increase of cytochrome C, caspase-3 and caspase-9 may be involved in this process.

## Background

5-Fluorouracil (5-FU) is one of the most common and effective clinical chemotherapy medications for treatment of digestive tract tumors, with a specific set of effects. Internally, 5-FU not only transforms into corresponding nucleotides and helps develop an anti-tumor effect, it can also induce apoptosis. Theoretically, 5-FU can bring about carcinogenic cell DNA deterioration and acceleration of tumor cell apoptosis [1,2]. Whereas 5-FU only had a single effective rate of 24%, and approximately 31% as the first-line drug of clinic chemotherapy on colon cancer, the increase in dosage could cause severe toxic- and side-effects, and gradually increasing drug resistance also limited its dosage and therapeutic effect [3,4].

Many epidemiology data, lab studies and clinical observations show that NSAIDs, nonsteroidal anti-inflammatory drugs, are effective at prevention and inhibition of digestive tract tumors, both as external and internal treatments. Cyclooxygenase-2 (COX-2) over-expressed is closely related to various tumors, especially gastrointestinal tumors [5]. Related studies have shown that the selective COX-2 inhibitor is an effective inhibitor of many kinds of organic tumors, including colon tumors, while also strengthening the effectiveness of chemotherapy treatment. At present, there are clinical reports of the effectiveness of combining selective COX-2 inhibitors with chemotherapy to treat digestive tract tumors; however, the exact mechanism of the anti-tumor effect created by this treatment is still unclear. Celecoxib, as a selective COX-2 inhibiter, holds a very good application prospect for the prevention and treatment of colon cancer. Meanwhile, it has been a hotspot of colon cancer research and receives more clinical attention due to its better gastrointestinal safety [6-8].

Celecoxib and 5-FU each have separate anti-tumor functions. Whether or not combined application of the two treatments can create a collaborative anti-tumor effect is still being debated [9-11]. For this reason, this experiment involves combined celecoxib and 5-FU treatment on a subcutaneous implantation tumor model of human colon cancer, in order to discover if such treatment does produce a collaborative anti-tumor effect and corresponding mechanism. These results may aid the progress and applicability of selective COX-2 inhibitors as a form of collaborative clinical treatment for tumor growth, and serve as the foundation for related future research.

## Methods

### Cell line and animal model

Human colon cancer HT-29 cells were purchased from the Cellular Biological Institute of the Science Academy of China in Shanghai and cultured in McCoy’s 5A with 10% fetal bovine serum (FBS). All cells were cultured at 37°C in humidified incubators with 5% CO_2_ and 95% air.

To establish the tumors in mice, HT-29 cells were grown in culture. Five- to six-week old athymic BALB/c nude mice (Animal Center of the Soochow University, China) were injected with 8 × 10^7^ HT-29 cells to activate tumor growth. After the tumor volume reached 50 to 100 mm^3^, the mice were randomly divided into four groups for further treatment as described below. This study conforms to the Guide for the Care and Use of Laboratory Animals published by the US National Institute of Health (NIH Publication No. 85–23, revised 1996) and was approved by the Moral and Ethical Committee of the First Affiliated Hospital of Soochow University.

### 5-FU and COX-2 inhibitor treatment

The selective COX-2 inhibitor, celecoxib (production NO. 639 T), was a generous gift of Pharmacia and Upjohn, Ltd. (Suzhou, China). The control group took in purified saline, the 5-FU group was given 5-FU (20 mg/kg), the celecoxib group was given celecoxib (1,500 ppm, 1.5 mg/ml), and the 5-FU + celecoxib group was given the combination of celecoxib and 5-FU (1,500 ppm celecoxib + 20 mg/kg 5-FU). All animals started to receive drug intervention after the subcutaneous xenograft model was established. The administration method of celecoxib was a fresh solution prepared by dissolving celecoxib in sterilized distilled water, and the nude mice took in the solution freely every day till the end of the experiment. 5-FU treatment was also administered via abdominal injection starting on the same day of celecoxib administration, and was continued for five days at a rate of one treatment per day. The dosage needed for each nude mouse was calculated according to the mouse’s weight, and fluid volume was adjusted to be injected into the nude mice, 1 ml for each mouse. The nude mice in the control group and the celecoxib group were given intraperitoneal injections upon group division for five successive days, once per day, and the menstruum was 1 ml normal saline. When all mice had successfully formed colorectal tumor masses, the length (a) and width (b) of the tumors were measured by electric vernier calipers every four days. The approximate volume of the tumor was calculated according to: V = a × b^2^/2. The nude mice were euthanized after 28 days of observation, and the tumor specimen was taken by dissection, and the approximate volume of the tumors was calculated. Finally, the tumor was weighed and the tumor inhibition rate (%) = ((the average weight of the tumors from the control group - the average weight of tumors from the treatment group)/ the average weight of tumors from the control group) × 100%.

### Histopathology

For histological examination, the stomach, intestine, liver and colon of the animals were excised and fixed in 10% neutral buffered formalin after the animals were sacrificed. Paraffin embedded sections (5 μm) were cut and stained with hematoxlin and eosin for histological examination.

### Immunohistochemisty for cytochrome C, caspase-3 and caspase-9 protein

Xenograft tumor tissue paraffin sections underwent routine deparaffinage, using gradient alcohol hydration, were sealed for 15 minutes with 1% H_2_O_2_, rinsed with 0.01 mol/L PBS. Antigens retrieval was carried out by microwave oven heating for 15 minutes. The sections were sealed in 2% normal goat blood serum for one hour, then, dropwise mouse anti-human caspase-3 and caspase-9 monoclonal first antibody (1:1,000; Santa Cruz Biotechnology, Santa Cruz, CA, USA), cytochrome C monoclonal antibody (1:500; Santa Cruz Biotechnology) were added, incubated overnight under 4°C, then a second antibody (1:200; Santa Cruz Biotechnology) marked by HRP was added dropwise after a phosphate-buffered saline (PBS) rinse. The tissue was then incubated at room temperature for 2 hours, colored with DAB for 15 minutes, and counterstained with hematoxylin. The results were judged positive as was previously described [12]. The evaluation criteria were as follows: (1) Staining intensity was evaluated and assigned a score of 0 to 3+: 0 = negative, 1 + = weak, 2+ = moderate, 3+ = strong. (2) Immunoreactivity score was assigned based on the proportion of positive cells over total cells (percent positivity) ranging from 0 to 100% on a scale of 0 to 3: 0 = 0% positive cells, 1 = 1 to 25% positive cells, 2 = 26 to 50% positive cells, 3 = >60% positive cells. The IHC scoring was then assigned by multiplying the staining score and immunoreactivity score, and the score thus ranged from 0 to 9.0.

### Transmission electron microscope

A total of 60 mg of xenograft tumor tissue mass was fixed in 2.5% glutaral for no less than two hours, prepared with saturated uranyl acetate with 70% alcohol. After 30 minutes’ stirring, the tissue was fixed with 4% osmic acid three times, then rinsed with phosphate buffered saline. The tissue was re-warmed with embedding material after being rinsed with 50% alcohol, then the sample was placed in a capsule and polymerized by heating (at 63°C for 72 h).Enteric-coated capsules were made after 48 hours, and then apoptosis was observed under an electron microscope.

### TUNEL assay

The percentage of apoptosis cells were detected by commercially available (terminal deoxynucleotidyl transferase dUTP nick end labeling) TUNEL assay kits (XiTang Biotechnology Corp., Shanghai, China), according to the instructions provided by the manufacturer. The karyotin of apoptosis nucleus was pitchy, while that of the non-apoptosis nucleus was calamine blue. Ten high power fields (HPF) were selected for each slice, and the number of positive cells was counted for every 1,000 cells. The apoptosis rate (%) = number of positive apoptosis cell/1,000 × 100%.

### Western blot analysis

Western blot was performed as described previously [13]. Membranes were developed using ECL reagents (Denville Scientific, Metuchen, NJ, USA) and exposed to X-ray films. Mouse original first antibody caspase-9 (1:1,000; Santa Cruz Biotechnology), mouse original first antibody caspase-3 (1:1,000; Santa Cruz Biotechnology) and mouse original first antibody COX-2 (1:1,000; Santa Cruz Biotechnology) were used for Western blot analysis.

### Statistical analysis

All data were presented as means ± SD. Statistical analysis was made by analysis of variance (ANOVA) followed by LSD-t *post-hoc* test for multiple comparisons using SPSS16.0 (SPSS Inc., Chicago, IL, USA). A *P*-value of <0.05 was considered to be statistically significant.

## Results

### General observations

**Figure 1:**
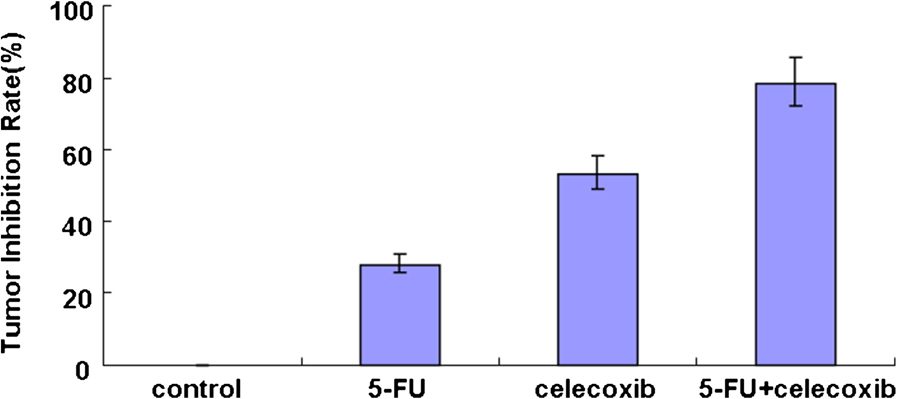
**Comparison of tumor inhibition rate in each group.** The tumor inhibition rate i the control group, the 5-FU group, the celecoxib group and the 5-FU + celecoxib group was 0%, 27.81%, 53.02% and 78.37%, respectively. *P* <0.05 among all groups.

**Figure 2:**
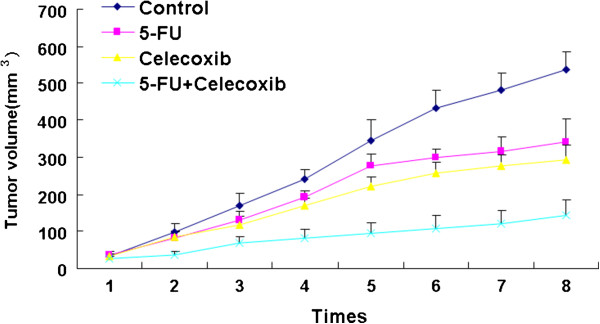
**The influence of 5-FU and celecoxib on tumor growth. **The anti-tumor effect on the 5-FU + celecoxib group was better than the other groups.

All animals successfully formed colorectal tumor masses before being sacrificed. Both the volume and the weight of tumors were significantly suppressed in the treated group compared with the control group (*P <0.05*) (Table 1). The tumor inhibition rate in the control group, 5-FU group, celecoxib group and 5-FU + celecoxib group was 0%, 27.81%, 53.02% and 78.37%, respectively, and the combined group had the highest tumor inhibition rate (*P* <0.05) (Figure 1). The effect of combined application of celecoxib and 5-FU for the treatment of colon carcinoma was better than for the other groups (Figure 2). No animal showed signs of liver metastasis, hyperemia, edema, erosion, bleeding or ulceration of the stomach and intestine mucosa; this was confirmed by histopathology.

**Table 1:** **The volume and weight of colon tumor**^**※ **^

**Group**	**Volume of tumor (cm**^**3**^**)**	**Weight of tumor (g)**
control	0.5367 ± 0.0532	0.5924 ± 0.0612
5-FU	0.3429 ± 0.0875^a^	0.4276 ± 0.0629^a^
celecoxib	0.1947 ± 0.0386^a^	0.2783 ± 0.0314^a^
5-FU + celecoxib	0.1428 ± 0.0236^a,b,c^	0.1281 ± 0.0219^a,b,c^

Note: ※ Values represent the mean ± SD; ^a ^There were significant differences in the volume and the weight of tumors between each treatment group compared with the control group, *P *<0.05. ^b ^Compared with the 5-FU group, the volume and the weight of tumors were dramatically lower in the 5-FU + celecoxib group, *P *<0.05. ^C ^Compared with the celecoxib group, the volume and the weight of tumors were also significantly lower in the 5-FU + celecoxib group, *P *<0.05.

### Apoptosis in the tumor cells

**Figure 3:**
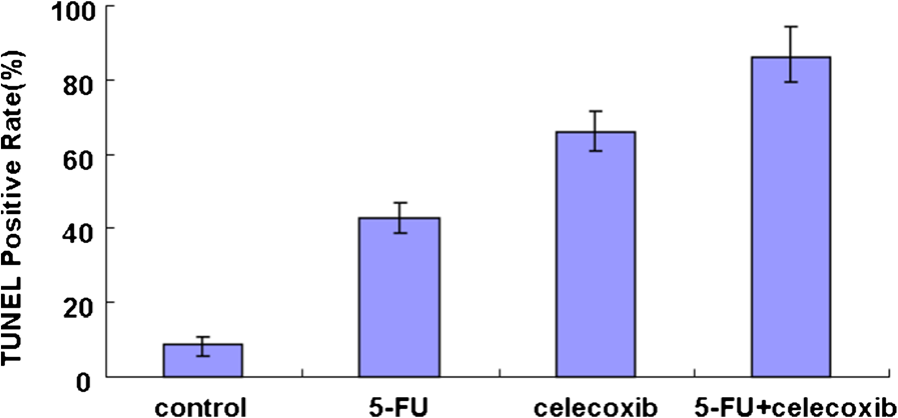
**Comparison of TUNEL positive rate in each group.** The rate of tumor apoptosis in the control group, 5-FU group, celecoxib group and 5-FU + celecoxib group was 9.5%, 47.5%, 68.5% and 80.1%, respectively, *P* <0.05 among all groups.

**Figure 4:**
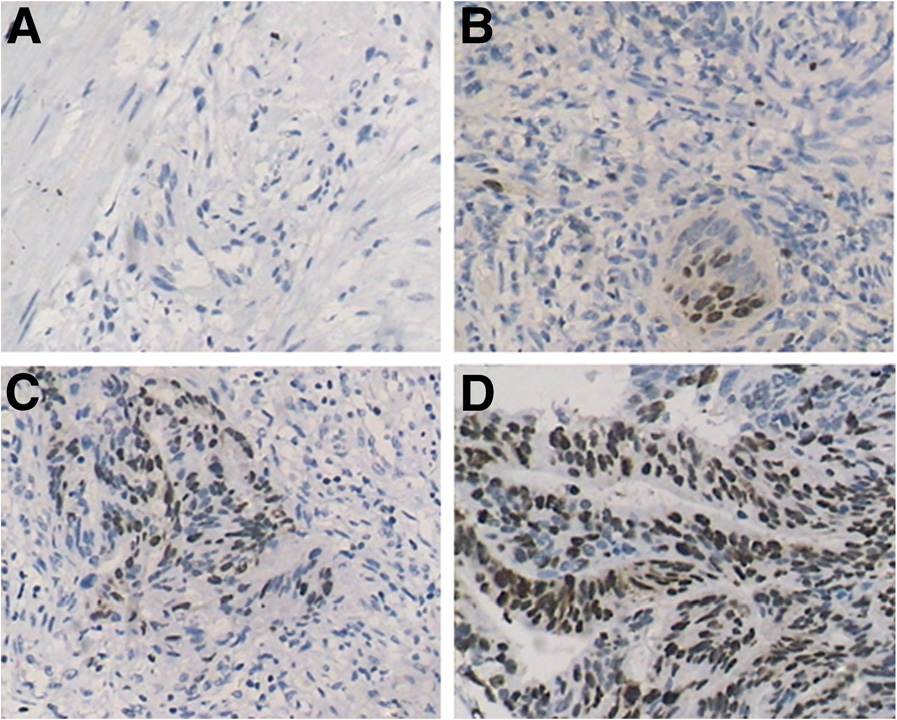
**Detection of apoptotic tumor cells by TUNEL assay.** TUNEL-positive cells (apoptotic cells) were stained black-brown (magnification: × 400). There were more apoptotic cells in the 5-FU + celecoxib group than in the other groups. (**A**, control group; **B**, 5-FU group; **C**, celecoxib group; **D**, 5-FU + celecoxib group).

**Figure 5:**
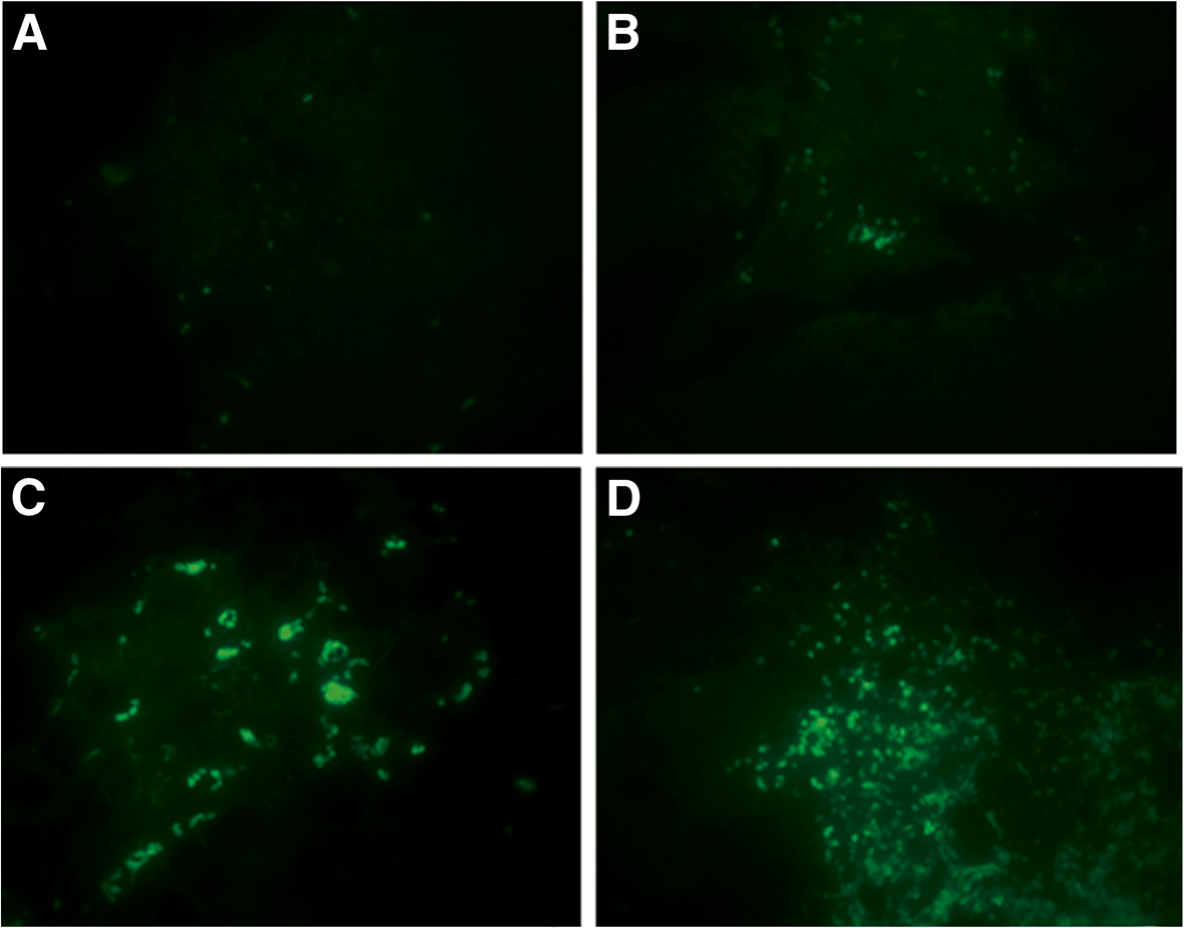
**Observation of apoptotic tumor cells by fluorescence microscope. **Apoptotic cells were stained green fluorescence (magnification: ×400). There were more apoptotic cells in the 5-FU + celecoxib group than in the other groups. (**A**, control group; **B**, 5-FU group; **C**, celecoxib group; **D**, 5-FU + celecoxib group).

**Figure 6:**
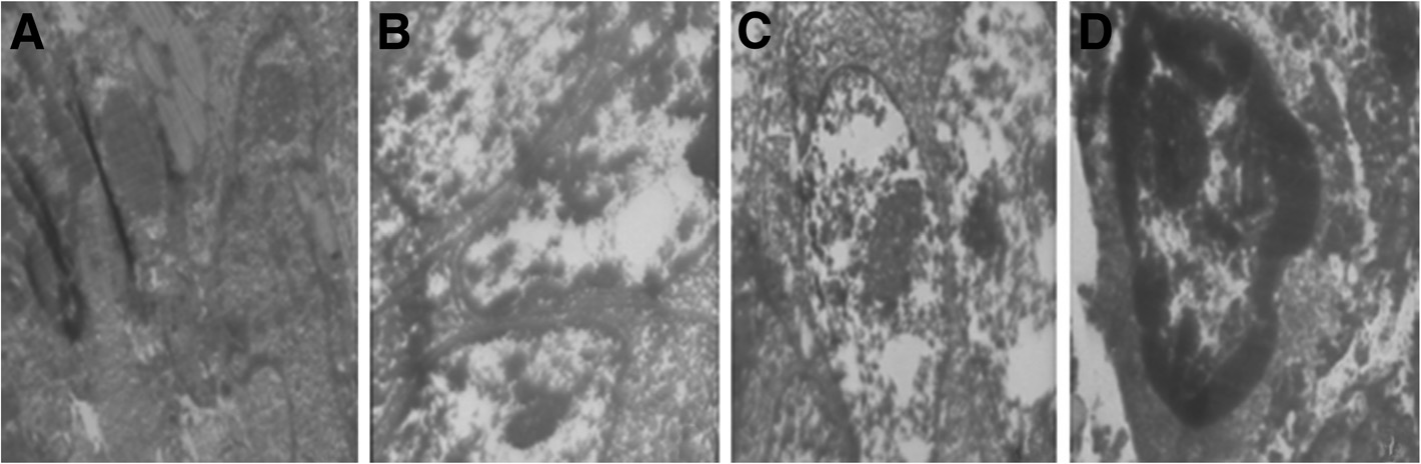
**Observation of apoptotic tumor cells by transmission electron microscope (Magnification: × 6000)**. The morphology alteration of apoptotic tumor cells in the 5-FU + celecoxib group was more typical than that in other groups. (**A**, control group; **B**, 5-FU group; **C**, celecoxib group; **D**, 5-FU + celecoxib group).

TUNEL detection showed that the number of apoptosis cells dramatically increased in treated mice compared with that in the control group; the rate of apoptosis was 47.5%, 68.5% and 80.1% in the 5-FU group, celecoxib group and 5-FU + celecoxib group, respectively, while only 9.5% in the control group (*P* <0.05) (Figures 3 and 4). The nucleus of apoptosis cells after dyeing in fluorescence microscope presents green fluorescence and was most obvious in the 5-FU + celecoxib group (Figure 5). Electron microscopy showed that in the treatment group the cellular outlines were extremely irregular, the cell body and nucleus decreased in size, nuclear membrane was intact, the nucleolar zone completely disappeared, chromatin was aggregated, became tight and concentrated beneath the nuclear membrane, part of mitochondria swelled and enlarged, the cell membrane was intact, nuclear fragmentation existed, apoptotic bodies encapsulated by membranes were scattered among the cells and contained residual nucleus and organelles (Figure 6). The apoptosis expression was especially typical in the 5-FU + celecoxib group, but was not demonstrated in the control group.

### Cytochrome C, caspase-3, caspase-9 and COX-2 protein expression

**Figure 7:**
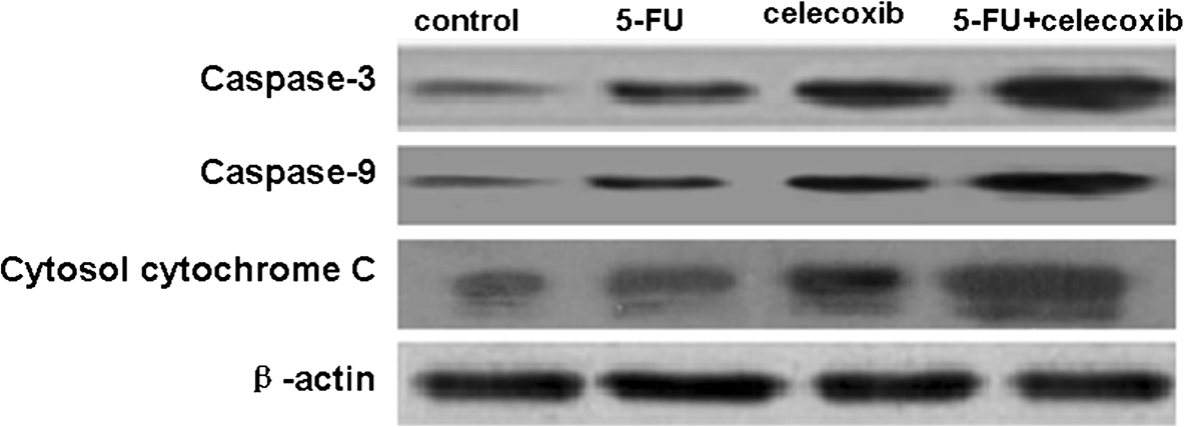
**Detection of protein expression of cytochrome C, caspase-3 and caspase-9 by Western blot. **The protein expression of cytochrome C, caspase-9 and caspase-3 in the 5-FU + celecoxib group was significantly stronger than that in other groups.

**Figure 8:**
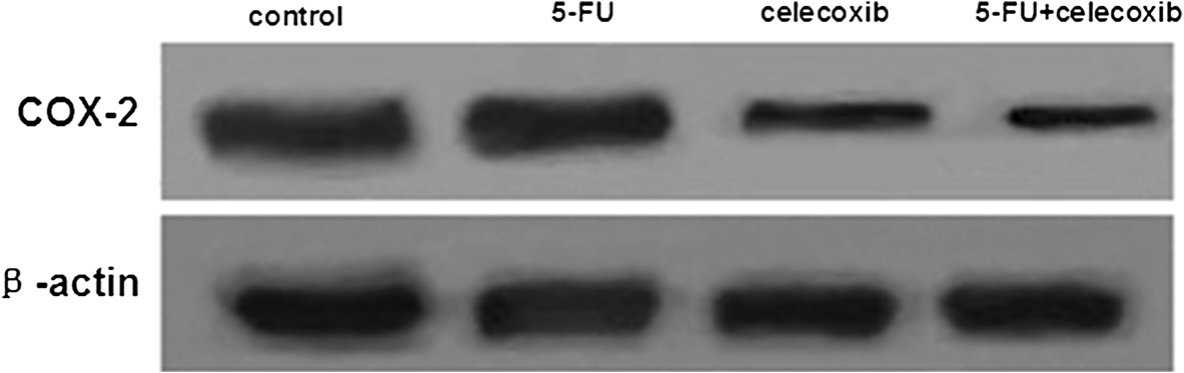
**Detection of protein expression of COX-2 by Western blot. **In the 5-FU group and 5-FU + celecoxib group, COX-2 expression was suppressed more than in the control group and celecoxib group. COX-2 expression was inhibited more obviously after the combined drug treatment.

Immunohistochemisty detection showed that the protein expression of cytochrome C, caspase-9 and caspase-3 was mainly in the apoptosis cells. In the apoptosis tumor cells, the staining of cytochrome C, caspase-9 and caspase-3 protein positive expression were mainly cytoplasm staining; a small part of the staining was nucleus and nuclear membrane staining and brownish yellow particles. Experimental results showed that the protein expression of cytochrome C, caspase-9 and caspase-3 in the interference group significantly increased compared with the control group (*P* <0.05) and more obviously in the drug combination group (the 5-FU + celecoxib group) (*P* <0.05, Table 2). Results were confirmed by Western blot (Figure 7). In the 5-FU group and the 5-FU + celecoxib group, COX-2 expression was more suppressed than that in the control group and celecoxib group. Also COX-2 expression was inhibited more obviously after combined drug treatment. While between the control group and the 5-FU group no obvious differences were observed (Figure 8).

**Table 2:** **Expression levels of cytochrome C, caspase-3 and caspase-9**^**※ **^

**Group**	**Cytochrome C**	**Caspase-9**	**Capase-3**
control	1.7542 ± 1.1869	2.1837 ± 1.3627	1.7562 ± 1.2761
5-FU	3.7397 ± 1.2963^a^	3.0527 ± 1.0667^a^	2.9242 ± 1.6728^a^
celecoxib	5.6286 ± 1.0629 ^a^	5.9782 ± 1.2869 ^a^	6.7197 ± 1.1869 ^a^
5-FU + celecoxib	7.9784 ± 1.1265 ^a,b,c^	7.7281 ± 1.0691 ^a,b,c^	7.9942 ± 1.0014 ^a,b,c^

Note: ※ Values represent the mean ± SD; ^a ^There were significant differences in the protein expression of cytochrome C, caspase-9 and caspase-3 between each treatment group compared with the control group, *P *<0.05. ^b ^Compared with the 5-FU group, the protein expression of cytochrome C, caspase-9 and caspase-3 was significantly higher in the 5-FU + celecoxib group, *P* <0.05. ^C ^Compared with the celecoxib group, the protein expression of cytochrome C, caspase-9 and caspase-3 was also significantly higher in the 5-FU + celecoxib group, *P *<0.05.

## Discussion

Colon cancer is one of the common malignant tumors in the digestive system, and the early diagnosis of colon cancer is very difficult. The main therapeutic methods of treating colon cancer are still radiotherapy and chemotherapy. As the first-line drug of clinical chemotherapy for colon cancer, 5-FU has not only poor efficacy, but also has significant side effects [3,4]. A therapeutic alliance could possibly improve the effect of chemotherapy on these tumors, and meanwhile reduce the adverse reaction of using a single drug.

Attention has recently been paid to un-cytotoxic drugs in tumor therapy research, and there have been many developments in the research of the COX-2 inhibitor’s effects on inhibiting tumor growth [14-17]. Celecoxib is a kind of highly selective COX-2 inhibitor, and has stronger pharmacologic actions and less adverse reactions compared with traditional NSAIDS [18]. Recent research has also found that selective COX-2 inhibitors can enhance the sensitivity of tumor cells to many kinds of chemotherapeutic drugs, thus reducing the IC_50_ of chemotherapeutic drugs by nearly 70% [19]. By using cancer cell lines cultured *in vitro* as research targets, and observing the effect of the selective COX-2 inhibitor nimesulide on various anti-tumor drugs (including DDP and VP16), Zheng [20] found that there were synergistic effects when nimesulide was used in combination with cytotoxic chemotherapeutic drugs. Our research indicated that the apoptotic morphology change was most significant and had the highest tumor inhibition rate in the drug combination group.

In order to further investigate the mechanism of celecoxib’s chemotherapy sensitization, we examined the protein expression of cytochrome C, caspase-9 and caspase-3. Our results showed that cytochrome C, caspase-9 and caspase-3 were up-regulated after treatment with celecoxib and 5-FU, especially in combination. This reveals that the combined use of two drugs had synergistic, anti-tumor effects, whose mechanism of action may be associated with the activation of cytochrome C-dependent apoptosis signal pathway. Some papers also reported that NSAIDs, such as indomethacin and sulindac, could suppress the transmembrane protein ATP dependent efflux pump (multidrug resistance associated protein, MRP) p2 glucoprotein related to chemotherapeutic efflux, and inhibit the activity of glutathione transferase. Through these pathways, NSAIDs could inhibit chemotherapeutic efflux and decrease the multiple drug resistance of tumor cells against chemotherapeutics [21]. In addition to animal experiments, there were some reports of the Phase I and Phase II clinical trials on celecoxib in combination with chemotherapy treatment for cancer [22-24]. Relevant test results indicated celecoxib has synergistic anti-tumor effects. A therapeutic alliance could reduce the dose and the adverse reaction of chemotherapy drugs. Some clinical research proved that combined application of celecoxib could effectively improve the three-year survival rate of patients with advanced colorectal cancer [25,26].

In our research, we also found that celecoxib combined with 5-FU could inhibit COX-2 protein expression. In recent years, much evidence has documented that COX-2 could up-regulate apoptosis suppressor genes’ bcl-2 levels by activating MAP kinase and bcl-2 could dramatically inhibit activation of cytochrome C pathway [27,28]. Our experiment showed that the synergistic anti-tumor effect with administration of 5-FU and celecoxib is not only related to improving the chemotherapy sensitization of 5-FU, but also may be associated with the effect by which 5-FU can enhance the ability of celecoxib to inhibit protein expression of COX-2. The chemotherapy agent 5-FU, which has been used against cancer for about 40 years, acted in several ways, but principally as a thymidylate synthase inhibitor. Interrupting the action of this enzyme blocked synthesis of the pyrimidine thymidine, which was a nucleoside required for DNA replication [2]. Administration of 5-FU caused a scarcity in dTMP, so rapidly dividing cancerous cells undergo cell death via thymineless death. According to the results of this experiment, we speculate that 5-FU can promote the effect that celecoxib inhibits the protein expression of COX-2 by regulating the gene expression.

Current research has shown that NSAIDs, including selective COX-2 inhibitors, could augment the sensitivity of many kinds of tumors toward chemotherapeutics, but its mechanism was still unknown, and it may involve multiple molecular mechanisms. Our experiment utilized a combination to implement drug interference based on the model of xenograft tumors in nude mice, and proved that when celecoxib and 5-FU are used in combination they had synergic anti-tumor effects. Suppression of COX-2 and activation of the apoptosis signal might be related to these processes.

## Abbreviations

5-FU: 5-fluorouracil; TUNEL: Terminal deoxynucleotidyl transferase dUTP nick end labeling; COX-2: Cyclooxygenase-2; NSAIDs: Nonsteroidal anti-inflammatory drugs; FBS: Fetal bovine serum; PBS: Phosphate-buffered saline; HPF: High power fields; ANOVA: Analysis of variance.

## Competing interests

The authors declare that they have no competing interests.

## Authors’ contributions

D-QZ, QG and J-HZ supervised the research project, participated in the data collection and drafted the manuscript. W-CC acted as corresponding author and did the revisions. D-QZ, QG and J-HZ contributed equally to this work. All authors read and approved the final manuscript.
